# The Impacts of Air Pollution on Mental Health: Evidence from the Chinese University Students

**DOI:** 10.3390/ijerph17186734

**Published:** 2020-09-16

**Authors:** Daqing Zu, Keyu Zhai, Yue Qiu, Pei Pei, Xiaoxian Zhu, Dongho Han

**Affiliations:** 1 School of Foreign Studies, China University of Mining and Technology (Xuzhou), Xuzhou 221116, China; dqzu@cumt.edu.cn; 2 Centre for Australian Studies, School of Foreign Studies, China University of Mining and Technology (Xuzhou), Xuzhou 221116, China; 3 School of English Culture and Literature, Beijing International Studies University, Beijing 100024, China; qiuyue@bisu.edu.cn; 4 School of Economics and Management, Tongji University, Shanghai 200092, China; pp@tongji.edu.cn; 5 Business School, Teesside University, Middlesbrough TS1 3BA, UK; x.zhu@tees.ac.uk; 6 Bartlett School of Planning, University College London, London WC1H 0NN, UK; dongho.han.13@ucl.ac.uk

**Keywords:** air pollution, mental health, well-being, life satisfaction, undergraduate students, China

## Abstract

A growing number of developing countries have experienced worsening air pollution, which has been shown to cause significant health problems. However, few studies have explored the impact of air pollution on the mental health of university students, particularly in the Chinese context. In order to address this gap, through a large-scale cross-sectional survey, this study aims to examine the effects of air pollution on final-year Chinese university undergraduates’ (due to graduate in 2020) mental health by employing multivariable logistic regression. Our findings show that, first, although normal air quality is not strongly associated with lower levels of negative mental health, there is a strong link between poor air quality and higher levels of negative mental health. More specifically, life satisfaction hedonic unhappiness and depression measured by the Centre for Epidemiological Studies’ Depression scale (CES-D) are statistically associated with air pollution. In addition, we also found that gender is a significant factor, as males had more than 1.6 times greater odds of increased mental health problems compared to their female counterparts. Place of birth also plays a significant role in participants’ mental health. Moreover, undergraduates with urban household registration experienced significant levels of hedonic unhappiness and depression on the CES-D scale. Finally, we found that there is an association between respondents’ economic situation and their mental health too. Overall, this study contributes to the research on air pollution management and mental health intervention, particularly in relation to student groups. The undergraduate curriculum should provide more guidance and suggestions on promoting mental health and establishing positive attitudes to life and academic study of the final year students, under the context of air pollution in China.

## 1. Introduction

A growing number of developing countries are experiencing serious air pollution problems [1]. For instance, as Greenstone and Hanna [2] noted, levels of air pollution in China are approximately six to eight times higher than those in the United States. Air pollution is directly related to health risks. Several Chinese cities have some of the worst air quality in the world, and 40% of all premature deaths caused by poor air quality in 2010 were in China [3]. It has been well documented that air pollution has resulted in more cardiovascular diseases [4,5], respiratory diseases [6,7], higher mortality [8] and poorer physical health [9,10]. However, little research has been undertaken into the less tangible impacts (e.g., psychological impacts) of air pollution on mental health. Additionally, most of the relevant studies were carried out in developed countries, such as the USA, rather than developing countries where air pollution levels are generally higher [11,12]. Only a few research touches the relationships between mental health and air pollution. For example, Zhang et al. explored the impact of air pollution on mental health status, depressive symptoms, moment-to-moment happiness, and evaluative happiness [11]. Based on a match of a longitudinal survey and local air quality and weather conditions, they make use of variations in exposure to air pollution for the same individuals over time. The analytical results show air pollution reduces hedonic happiness and contribute to the increased rates of depressive symptoms [11]. However, what still remains unclear is how air pollution impairs mental health of the final year university students in China.

Previous studies evaluating the effects of air pollution have not paid much attention to the less tangible aspects, such as emotional and mental health generally, and the Chinese context in particular has been neglected [11,13,14]. The existing research investigates the tangible aspects such as physical health, so there is a research gap in which the mental health problems resulted from air pollution are not noticed. For example, Wang et al. [12], from the perspective of the social capital, investigated the relationship between air pollution and psychological depression in China. Moreover, only a very few studies have observed that university students’ mental health is impacted by air pollution. Therefore, this study attempts to fill the research gap, by focusing on the relationship between final-year university undergraduates’ mental health and air pollution.

Thus, the aim of this study is to examine the impacts of air pollution on Chinese university undergraduates’ mental health. The data were collected via a large-scale cross-sectional survey, comprising 4721 questionnaires completed by the final-year Chinese students (the subjects of this study were Chinese undergraduates due to graduate in 2020, comprising a total of 4721 participants from different cities and universities). Multivariable logistic regression was then used to assess the impact of air pollution on these students’ mental health. This study contributes to the current literature in the following ways. First, it assesses how undergraduates’ mental health is affected by air pollution. Unlike students at other levels and stages of education, final-year students are more likely to face difficult transitions from being a university student to adulthood and have to adapt to rapid social changes and challenges in their lives. Given that many Chinese university students are experiencing mental health problems [15], this paper could contribute to new empirical knowledge about final-year undergraduates’ mental health and the factors that affect it, as well as providing recommendations for improving students’ mental health. Second, this paper addresses air pollution from a different perspective and provides new insights for air pollution management and health interventions among the student population.

The paper is structured as follows. Following the introduction, the second section reviews the existing literature and then identifies the research gap. The third section describes the data and methods used. The analytical results are provided in the fourth section, while the final section comprises a discussion and conclusions.

## 2. Literature Review

A plethora of research has evidenced that mental disorders are associated with environmental changes [16,17,18]. According to a self-assessed mental health survey, Xue et al. [16] reported that an increase in air pollution is associated with a higher probability of self-assessed mental health problems. Similarly, Kioumourtzoglou et al. [19] also confirmed a significant association between higher levels of Inhalable fine and coarse particulate matter (PM_2.5_, PM_10_) and increased mental health problems. Even in cases of relatively low levels of PM_10_ air pollution, exposure to it has significant negative impacts on subjective life satisfaction and well-being [20]. In addition, long term exposure to higher levels of air pollutants (PM_2.5_, PM_10_, NO_2_) has been found to results in lower levels of happiness among adolescents [7]. Furthermore, sensitivity analyses have shown that aspects like the number of participants in the sample do not affect the relationship between air pollutants and adolescents’ happiness. Emerging research has recently started to explore the less tangible effects of air pollution, but currently there is uncertainty about the relative effects of different drivers [16].

Employing large-scale national surveys, some studies have explored the impacts of air pollution on mental health in China. Drawing on a nationwide longitudinal survey, Zhang et al. [11] evidenced that air pollution directly and significantly reduces hedonic happiness and increases the possibility of depressive symptoms, a finding which has received widespread public attention. Similarly, based on a nationwide population-based cross-sectional study, Shin et al. [14] investigated the association between ambient air pollutants and mental health status. They evidenced that air pollution is a risk factor for a wide range of potential mental health disorders. Moreover, participants’ socio-economic demographics were also considered in this study. In contrast to most of the existing literature, Chen et al. [13] focused on a specific group and quantified the causal effects of air pollution on the health status of Chinese adolescent students and their school attendance. Based on the data from the Guangzhou Centre for Disease Control and Prevention (CDC), they demonstrated a sizable deleterious effect of air pollution on school attendance. The harmful impacts were found to be non-negligible, even though the level of pollution was below the official criteria used to measure air quality. Thus, in China, ambient air quality standards cannot work to protect students. In addition, the relative effects of multiple environmental factors including air quality, residential greenness, mean temperature and temperature variability remain uncertain [16]. It has been argued that reducing air pollution emissions could improve people’s mental health in China. Specifically, an increase in mental health problems is significantly associated with increases in PM_2.5_ and changes of lifestyle often linked to urbanisation [16]. Furthermore, the data collected from local areas at city level (such as Beijing or Tianjin) may result in differences in the research results between different cities, which may derive from the heterogeneity of study populations and different research designs [16]. Therefore, the national survey used in this study needs to reconsider the representative exposure–response curves among the general population.

In addition, demographic data is used to study the mental health of individuals. Wang et al. [12] applied a social capital approach to studying the relationship between air pollution and psychological depression in China. For example, people who have higher levels of education and income are less likely to suffer from depression issues compared to those who are less educated and less affluent [21]. Wang et al. [12] extended the application of the social capital perspective to studying depression and noticed that social capital decreased the negative effects of PM_2.5_ concentration on depression among participants. Social capital (we follow the definition used in Kawachi et al. [22] research which sees social capital as a resource for providing individuals with greater convenience, and which is essential for the maintenance of a population’s health) is therefore confirmed to constitute a protective factor in the relationship between health and environmental hazards. From a labour market perspective, Zheng et al. [23] examined the impact of air pollution on the occupational location of university graduates and reported on the differences between undergraduates and postgraduates. Air pollution was proved to have differing impacts on individuals with different levels of educational attainment. Therefore, participants’ demographic information should also be taken into account when studying mental health.

Although emerging research has started to explore the relationship between mental health and air pollution, the mental health of young adults has been neglected. Final-year undergraduates are more likely to experience a critical transition from being a university student to adulthood, which brings plenty of challenges [24]. More importantly, a large proportion of these young adults suffer from serious sleep problems and mental health problems which are caused by environmental pollution [25]. Considering the large number of Chinese final-year undergraduates who are going through this transitional stage and experiencing mental health problems, studying the state of their mental health is crucial. Existing research has found that environmental pollution has a dramatic influence on mental health [25] and individual happiness and development [7]. However, it does not specifically focus on how final-year undergraduates’ mental health is impacted by air pollution. In order to fill this research gap, this study takes final-year undergraduates as its subject and investigates the effects of air pollution on their mental health.

## 3. Materials and Methods

### 3.1. Study Sample

We carried out an online survey to collect cross-sectional data relating to the self-assessed mental health of the final-year undergraduates. This study was approved by the Ethics Committee of School of Foreign Studies of China University of Mining and Technology on 1st January 2020 (No.20191128-001). The data collection took two and a half months, and in order to ensure its validity, we employed a random strategy for sending out the questionnaires to suitable participants. To meet the eligibility criteria, the participants had to be final-year undergraduate students and expected to graduate in June 2020. The questionnaires were distributed via WeChat. Suitable participants were identified and invited to take part at random and contacted through WeChat. They then had to complete an online survey designed to understand the impacts of air pollution on their mental health. Before filling in the questionnaire, participants were informed about the ethical issues and presented with a consent form. We eventually collected 5177 questionnaires in total, of which 4721 were valid for sequential analysis. Based on the data, we found that the participants came from 200 different universities, covering different levels of university: 985 project (the 985 project refers to the top 39 universities in China. The 985 project was proposed in 1998 by the Ministry of Education in the ‘Action Plan for Education Revitalisation for the 21st Century’ to provide generous funding and resources for selected higher education institutions that had the potential to deliver world-class research excellence), 211 project (the 211 project refers to the top 100 universities in China in the 21st century. The 211 project was launched by the State Council in 1995 in a bid to establish 100 world-class universities), Yiben (Yiben refers to the first tier of a bachelor’s degree), Erben (Erben refers to the second tier of a bachelor’s degree), Sanben (Sanben refers to the third tier of a bachelor’s degree)) and had a good gender balance. These participants studied their undergraduate subjects in 200 different universities located in 83 different cities in China. These cities included first-tier cities (Beijing, Shanghai, Guangzhou) and capitals of provinces and prefecture-level cities.

The average completion time for the online surveys was 12 min per survey. The questionnaire was supported by Wenjuanxing (professional survey software) and was sent to participants in the form of an online questionnaire via WeChat. In order to attract more respondents, we gave each participant a voucher worth between 1 and 3 RMB. The value of the voucher was randomly generated by the WeChat system. Receiving a voucher also encouraged the respondents to complete all the questions and thus enhanced the completion quality: as each ID only had one chance to get a voucher, this ensured that participants were only one given opportunity to complete the questionnaire. The voucher was sent out automatically by the Wenjuanxin website and the participant data was anonymised. Accepting a voucher did not require participants to provide personal information.

### 3.2. Data

This study used the China Family Panel Studies (CFPS) to investigate and measure the students’ mental health. CFPS is a comprehensive survey involving individuals, families and communities, and was compiled by Peking University [11]. The survey consists of data on the educational outcomes, family dynamics and relationships, economic activities, self-rated health, and subjective well-being of respondents. We therefore conducted the same survey with Chinese final-year university students. Existing studies have identified the advantages of using the CFPS, including geographical locations and dates, and multiple-level effects [26]. In relation to our research topic, the survey has the following advantages. First, the measurement scale not only assesses moment-to-moment happiness and mental well-being, but also long-term life satisfaction, which can help to measure the impact of pollution on mental well-being in a more comprehensive way. In addition, using longitudinal data allows unobserved individual factors which would otherwise bias the results to be eliminated [11].

Following the existing literature, we selected the following three kinds of mental health measure for our study: life satisfaction [20,27,28], hedonic unhappiness [11,29], and the Centre for Epidemiological Studies’ Depression scale (CES-D) [30]. The three key dimensions can reflect the participants’ mental health in a sound way [11]. The three dimensions consist of the most concerns of the final year university students. Life satisfaction (an evaluation indicator of subjective well-being) reflects the degree to which people’s own experience matches their long-term wishes and expectations for life as a whole [29]. There are many determinants of life satisfaction, including education, income and social status. In order to assess their level of life satisfaction, the respondents were required to answer the following question based on the CFPS: “how satisfied are you with your life overall?”. The values that they could select for their answers ranged from 1 (not satisfied at all) to 5 (very satisfied). In addition, we also measured hedonic unhappiness using the CFPS survey. The respondents were asked to answer the following question: to what degree have you found it difficult to cheer up during the past month? The answer scores ranged from 0 (never) to 4 (every day), and low values indicate a high degree of happiness. The CES-D is a self-reported scale measure used to reflect the degree of depressive symptomatology, and it focuses on individuals’ multidimensional emotional experiences over a shorter time period [30]. Moreover, the CES-D contains enough psychometric properties to be used for the specific and sensitive detection of depressive disorders [31]. The scale consists of six items, and each item has five options for respondents to select from. The scores for each option range from 0 (never) to 4 (every day). Thus, the final scores of the CES-D range between 0 and 24, and higher scores mean that respondents had experienced more negative symptoms in the past month. Based on the work of Burnam et al. [32] and Zhang et al. [11], we chose a binary indicator of depressive symptoms by using a cut-off point of four in our study.

The study took the air quality index (AQI) as a measure of air pollution. The AQI monitors PM_2.5_, PM_10_, SO_2_, and NO_2_ and can thus comprehensively reflect the state of air quality. The Ministry of Environmental Protection of China reports daily AQI observations at an urban level. In order to match the measurement of air pollution to those used to rate mental health, the air pollution data were standardised through demeaning (subtracting the mean from its every value) and then respectively dividing them by their own standard deviation. The estimated coefficient of air pollution can be regarded as the variation in an outcome variable (mental health) with respect to the change in air pollution by one standard deviation. Jiang et al. [33] provided a good example of an application of AQI to outdoor air pollution research and offered strategies for assessing air quality. We also followed Jiang et al.’s [33] methods and divided AQI into two broad categories: no pollution (excellent or good level); and pollution (light/moderate/high/serious pollution).

In addition, this study also observed the socio-demographic and health characteristics of respondents, as these may affect their experiences and perceptions of metal health. In this study, the socio-demographic covariates were age [34], gender, monthly living expenses [12], educational level [35,36], place of birth [37], and subjects studied at undergraduate level. Moreover, the study controlled for the self-rated health of respondents and whether they had any instrumental daily living activities and/or limitations in relation to these activities, because prior research has indicated that these factors affect perceptions of mental health and well-being [38].

### 3.3. Analytical Strategy

Due to the aforementioned socio-economic demographic variables, this study employed a multivariable model, because it has the capacity to include both continuous variables and categorical variables [39]. The categorical variables were measured on the same scale, ranging from 1 to 5. In order to ensure their validity, we also carried out T-tests to evaluate the sample mean differences for the continuous variables, and Chi-square tests for the categorical variables. In order to meet the assumptions of the analytical models, we controlled for collinearity between all the covariates. Importantly, we found that multicollinearity issues among independent variables were not a problem, because the largest Pearson’s correlation coefficient was 0.575. In addition, the models of ordered logistic regression were shown to meet the proportional odds assumption, which is indicated by the non-significant *p*-values.

Our results will be shown by two forms. That is, based on a dichotomous and three-level ordered categorical scale of the dependent variable in our study, bivariable and multivariable associations between every independent variable and dependent variable will be evaluated by employing logistic regression and ordered logistic regression. In our multivariable models, regardless of the significance level of bivariable analysis, the study makes a priori adjustment for all sociodemographic covariates. A two-tailed test (∝ =0.05) for statistical significance was defined. With the help of Stata 16 software, we analysed all the collected data. Although the multivariable model contains normal air quality and poor air quality in a same model, each of these independent variables are examined in different models so as to triangulate findings. Based on the method used by Primack et al. [40], we followed the three steps of sensitivity analysis to ensure the robustness of the research results. The first step involved conducting continuous variable analysis; second, the existing literature shows that a *p* value of less than 0.20 can avoid overcontrolling [40], so we used covariates with a *p* value of less than 0.20 to analyse the data; third, we carried out the analysis based on the psychological well-being cut-offs, so that we could ensure the research results were consistent with the first model. Because the results of the primary and additional models were similar, only the research results and analysis of the primary model are shown.

## 4. Results

Table 1 displays the descriptive analysis of the variables. We measured the mean differences of the continuous variables and the categorical variables using T-tests and Chi-square tests respectively.

These recognised the variance inflation factor (VIF) (average VIF is 1.29), indicating that multicollinearity issues among each independent variables in our models will not lead to great impacts on our results. psychological well-being is significant. Table 1 shows that the gender distribution of the sample is relatively well balanced (Female = 48%; Male = 52%). The age of the survey respondents ranged from 18 to 30 years old, and the mean was 22.6, with the Standard Deviation (SD) of 3.2. Participants whose monthly expenses ranged from 2001 to 3000 yuan accounted for 74% of the sample, and 58% of the participants were from urban areas. In terms of the subjects studied, undergraduates studying engineering accounted for the largest proportion (38%), while 32% were studying science and humanities and 30% were studying social science.

We operationalised mental health in tertiles, and the variables shown in Table 2 (AQI, PM_2.5_, PM_10_, SO_2_, NO_2_) demonstrated the significance of the relationship to mental health. The internal consistency of the four items was high (∝ =0.92). According to the analytical results, they were skewed to the right, which is indicated by a mean value of 7.63 (SD = 3.8) and a median of 7.2 (IQR = 5–20). Approximately 33% of respondents had low levels of life satisfaction but a similar percentage (34%) were at the opposite end of the scale within high levels of life satisfaction. Meanwhile, approximately 38% of the sample reported low levels of hedonic unhappiness, while 41% reported high levels. With regard to depression, about 39% of the sample reported low levels according to the CES-D scale, while about 37% reported high levels. Additionally, we found that the distribution of all the independent variables was skewed. Around 55% (SD = 31.63) of participants reported good air quality, with a median of 53% (IQR = 25–58), but over 69% of participants reported poor air quality and high levels of pollution, with a median of 57% (IQR = 24–76).

We established three groups of models for life satisfaction, hedonic unhappiness and depression on the CES-D scale respectively (see Table 3). Every first set of multivariable models investigated the relationships with mental health as a dichotomous variable. Model 1 shows good quality for every category (life satisfaction, hedonic unhappiness and CES-D), and Model 2 shows poor quality. Model 3 contains both of these independent variables. In addition, Table 3 includes all the covariates. In these models, AOR means the adjusted odds ratio and CI denotes the confidence interval. Regarding life satisfaction, in the case of Model 1, each 10% increase in normal air quality resulted in a 13% increase in life satisfaction (AOR = 1.13; 95%CI = 0.89–1.22). For Model 2, every 10% increase in poor air quality led to a 9% decrease in life satisfaction (AOR = 0.91; 95%CI = 0.80–1.33). Model 3 included both the independent variables in the same model and produced a similar result: for each 10% increase in normal air quality there was a 6% increase in life satisfaction, while each 10% increase in poor air quality was associated with a 2% decrease in life satisfaction. With regards to hedonic unhappiness, in Model 1, each 10% increase was accompanied by a 31% decrease in hedonic unhappiness (AOR = 0.69; 95%CI = 0.87–1.03). In the case of Model 2, a 10% increase in poor air quality resulted in an 8% increase in hedonic unhappiness (AOR = 1.08; 95%CI = 1.01–1.29). Model 3 included both these independent variables in the same model and reported a similar result. Similarly, in respect of CES-D, the first model showed that each 10% increase in normal air quality resulted in a 25% decrease in CES-D, while Model 2 revealed a 21% increase in CES-D.

Table 4 shows the second group of multivariable models that explored the relationships with mental health operationalised in tertiles. There are three models for every variable (life satisfaction, hedonic unhappiness and depression on the CES-D scale). Model 1 included normal air quality, Model 2 included poor air quality and Model 3 included both these independent variables. The second group of multivariable models produced similar results to the first group of multivariable models. All three models showed that gender (male) plays a significant role with regards to life satisfaction.

In the case of Model 1, both gender (male) and subject (engineering) have significant effects on life satisfaction. In Model 2, both gender (male) and subject (science) were also shown to significantly affect participants’ life satisfaction. In Model 3, gender (male) and subject (engineering) were both significant factors. In respect of hedonic unhappiness, monthly living expenses (3001 yuan) and place of birth (urban) were revealed to be key factors in all three models. In the case of Model 2, monthly living expenses (2001–3000 yuan) were shown to have a significant effect, while in Model 3, gender (male) was revealed to play a significant role. In relation to the CES-D scale, gender (male), monthly living expenses (2001–3000; 3001 yuan), education (Yiben; Sanben) and place of birth (urban) were all shown to have a statistically significant effect on depression.

## 5. Discussion

The differing demographic factors and differences in air quality that the final-year undergraduates experienced affected their mental health in different ways. Both positive and negative perceptions and self-reported feelings tended to influence their mental health in different ways, because individuals may be faced with widely differing situations during their last year of university. If they are not under too much pressure and feel that their career prospects and financial situation look encouraging, they may enjoy good mental health. However, many undergraduates who are trying to cope with considerable pressures may have significantly greater chances of suffering mental health problems.

This study confirmed the negative effects of air pollution on university students’ lives and academic work and modelled the association between air pollution and mental health. Good air quality is important for all human beings, but poor air quality can result in serious mental health problems. For example, regarding the multivariable associations of psychological well-being as a dichotomous variable (Table 3), when normal air quality increases 10%, the life satisfaction would increase 6%, whereas 10% increase of poor air quality can lead to 2% decrease in life satisfaction. Similarly, 1% increase in poor air quality is accompanied by 0.8% growth in hedonic unhappiness. In terms of CES-D, each 10% increase in normal air quality will lead to 25% and 21% decrease in models 1 and 2 respectively. The trend still holds in multivariable associations of psychological well-being operationalised in tertiles. Moreover, life satisfaction, hedonic unhappiness and CES-D scale are closely associated with air pollution. For instance, in multivariable associations of psychological well-being operationalised in tertiles, except Model 3 of life satisfaction, the results of normal air quality are significant with respect to life satisfaction, hedonic unhappiness and CES-D; similarly, most results of models in poor air quality are significant with psychological well-being. Overall, in all of our models, every increase in good air quality contributed to an increase in life satisfaction, and a decrease in hedonic unhappiness and depression.

The impacts of air pollution on final-year undergraduates’ mental health are influenced by various factors, including socioeconomic factors [12,23], and academic and financial pressures [24]. In this paper, we also found a series of significant variables of gender, age, place of birth, education, monthly living expense and university subject, which play an influential effect on final-year undergraduates’ mental health. For example, we confirmed that gender is a significant factor and demonstrated that male respondents had more than 1.6 times greater odds of increased mental health problems than their female counterparts. In China, although males are more likely to go university and earn higher incomes than the females, male students tend to be less happy due to the higher expectations and greater pressures that they face [41]. In addition, age is also a significant variable in Model 2 and 3 of CES-D. Secondly, place of birth plays a significant role in participants’ mental health. Undergraduates with urban registration had significant levels of hedonic unhappiness and depression. Lastly, we also found that there was an association between respondents’ economic situation and their mental health. Specifically, a higher level of monthly living expenses contributed to significantly greater life satisfaction and less unhappiness and depression. monthly living expense (2001–3000, 3001) has significant role in Model 2 and 3 of CES-D.

This study investigates the mental health of the final year university students in China, with a focus in the Chinese context. We studied a series of variables affecting mental health of final year students in Chinese universities, and these factors provided reference for other contexts to study the final year university students. The final year students around the world will and are experiencing transitions and pressure. For other contexts, characters in a specific context should be considered when the questionnaire is designed, because the air quality in different contexts is rather different. More importantly, the research design can also be adopted to study other contexts, for example, some developing countries who are experiencing severe air pollution. As for other contexts such as south Europe and south America, the research design of studying university students can be adopted and then adapted to the different contexts. In this study, the variables regarding undergraduate students include living costs, university tier, subjects, which can be used to study the final year students in other contexts. We employed logistic regression and ordered logistic regression to measure the impacts of air pollution on mental health, which can be an example for studying the mental health of the university students.

## 6. Conclusions

This paper studied final-year undergraduates in China and found that normal air quality was not strongly associated with lower levels of negative mental health, but poor air quality had strong links to higher levels of negative mental health. As a special group experiencing challenging transitions from university life to adulthood, and dealing with considerable academic and financial pressures, final-year university students are more likely to suffer from mental disorders than their peers [24]. This study contributes to our understanding of final-year undergraduates who are experiencing significant transitions in life and it is useful to know what kind of associations there are between air quality and mental health. We also aimed to provide suggestions and reflections for final-year undergraduates to help them improve their mental health. Although in 2005, the Chinese government released “the Opinion about Strengthening and Improving College Students’ Ideological and Political Education”, which puts extensive emphasis on the college mental health education and consultation services [42], a large proportion of university students are meeting mental health issues resulted from air pollution. In current situation, Chinese government should design the political and mental health curriculum based on the new challenges such as air pollution and COVID-19. The university curriculum should provide more guidance for improving the mental health of the final year students and leading students to establish positive attitudes to life and academic study. More practical interventions can be introduced to improve mental health of the final year students.

This study has some limitations, the main one being the use of cross-sectional data. Therefore, a well-designed longitudinal study would be highly recommended for future research. Air pollution is not a steady variable and a longitudinal study would be able to ensure that the analytical results were more precise. The research findings could be useful for mental health practitioners working with university students. In order to provide effective suggestions to improve their mental health, specific strategies are required to improve air quality generally and ensure that the air quality is good in universities. In addition, due to the variations in respondents’ individual situations, the socio-economic characteristics of final-year students should also be taken into account.

## Figures and Tables

**Table 1:** Descriptive statistics (n = 4721).

Variables	Mean/Sample %	SD	Test Statistics ^1^	*p*-Value ^2^
Mental Health				
Life satisfaction	2.4	1.1	t = 0.47	*p* = 0.003
Hedonic unhappiness	0.8	0.9	t = 3.21	*p* = 0.004
CES-D	2.9	3.7	t = 0.32	*p* = 0.001
Air Pollution				
AQI	99.2	61.1	t = 0.37	*p* = 0.011
Age			t = 4.39	*p* = 0.025
18	3%			
19–20	5%			
21–24	86%			
25–30	6%			
Gender				
Female	48%		X2 = 4.22	*p* = 0.018
Male	52%			
Monthly living expenses (Chinese Yuan)				
1000	3%		X2 = 5.13	*p* = 0.000
1001–2000	14%			
2001–3000	74%			
3001	9%			
Education				
985 project	12%		X2 = 1.76	*p* = 0.012
211 project	19%			
Yi Ben	21%			
Er Ben	33%			
San Ben	15%			
Place of birth				
Rural	42%		X2 = 1.69	*p* = 0.007
Urban	58%			
Subjects				
Humanities and Social Sciences	32%		X2 = 1.57	*p* = 0.015
Science	30%			
Engineering	38%			

^1^ Test-statistics means t-test which assesses the two-sample mean differences of variables. Chi-test assesses the categorical variables (gender, education and etc.). ^2^ *p* refers to the *p*-value, suggesting the significance in statistics.

**Table 2:** Whole sample characteristics and bivariable associations.

	Life Satisfaction			*p*-Value	Hedonic Unhappiness			*p*-Value	CES-D			*p*-Value
	High (20)	Medium (17–19)	Low (5–16)		High (20)	Medium (17–19)	Low (5–16)		High (20)	Medium (17–19)	Low (5–16)	
Air Pollution												
Normal air quality mean (SD)	61(34.12)	53 (27.69)	50 (33.07)	0.000	53 (41.02)	55 (27.29)	56 (26.57)	0.001	62 (37.22)	53 (32.19)	49 (25.47)	0.000
Normal air quality, median (IQR) ^1^	57 (32,64)	52 (26, 57)	49 (17, 53)	0.001	59 (34, 62)	53 (28,57)	46 (13, 55)	0.000	61 (33, 68)	55 (29, 59)	42 (13, 47)	0.000
Poor air quality mean (SD)	72 (39.24)	69 (35.15)	65 (36.66)	0.003	73 (42.07)	69 (38.19)	64 (30.79)	0.002	83 (42.29)	69 (39.78)	54 (28.98)	0.001
Poor air quality, median (IQR)	62 (24, 83)	57 (30, 73)	53 (19, 71)	0.005	69 (35, 86)	54 (24, 84)	49 (14, 57)	0.001	42 (17, 64)	53 (24, 78)	77 (32, 85)	0.003
Age				0.027	0.094							0.038
18	13(1)	61(4)	17(1)		33(2)	21(1)	37(3)		13(1)	27(1)	51(4)	
19–20	80(6)	71(5)	29(2)		69(5)	17(1)	94(6)		61(4)	39(2)	80(6)	
21–24	981(73)	1212(80)	1377(74)		889(65)	1432(75)	1249(85)		1356(81)	1312(72)	902(73)	
25–30	238(18)	189(12)	439(23)		361(27)	427(23)	78(5)		243(15)	432(24)	191(16)	
Gender			0.213		0.031							
Female	698(53)	899(57)	550(30)		778(58)	996(53)	373(25)		932(56)	361(20)	854(69)	
Male	628(47)	634(43)	1312(70)		574(42)	901(47)	1099(75)		741(44)	1449(80)	384(31)	
Monthly living expenses (Yuan)	0.001				0.009							
1000	26(2)	316(20)	694(37)		592(44)	330(17)	114(8)		732(44)	122(7)	182(15)	
1001–2000	691(52)	892(58)	912(49)		528(39)	1358(72)	609(41)		405(24)	1277(71)	813(65)	
2001–3000	488(36)	228(15)	217(11)		112(8)	152(8)	669(45)		378(23)	321(18)	234(19)	
3001	121(9)	97(6)	39(2)		120(9)	57(3)	80(5)		158(9)	90(4)	9(1)	
Education		0.003			0.017				0.322			
985 project	261(20)	492(32)	197(11)		52(4)	395(21)	503(34)		637(38)	177(10)	136(11)	
211 project	733(55)	503(33)	132(7)		173(13)	735(39)	460(31)		559(33)	491(27)	318(26)	
Yi Ben	226(17)	229(15)	384(21)		312(23)	326(17)	201(14)		71(4)	227(13)	541(44)	
Er Ben	71(5)	131(8)	698(37)		393(29)	263(14)	244(17)		216(13)	485(27)	199(16)	
San Ben	35(2)	178(11)	451(24)		422(31)	178(9)	64(4)		190(11)	430(23)	44(3)	
Place of birth		0.031			0.217							
Rural	548(41)	891(58)	901(48)		713(53)	971(51)	656(45)		656(39)	706(39)	978(79)	
Urban	778(59)	642(42)	961(52)		639(47)	926(49)	816(55)		1017(61)	1104(61)	260(21)	
Subjects		0.018			0.038				0.007			
Humanities and Social Sciences	312(24)	634(41)	367(20)		532(39)	419(22)	362(25)		793(47)	233(13)	287(23)	
Science	178(13)	229(15)	824(44)		519(38)	504(27)	208(14)		339(20)	573(32)	319(26)	
Engineering	836(63)	670(43)	671(36)		301(22)	974(51)	902(61)		541(32)	1004(55)	632(51)	

^1^ IQR means inter-quartile range.

**Table 3:** Multivariable associations of psychological well-being as a dichotomous variable.

	Life Satisfaction			Hedonic Unhappiness			CES-D		
	Model 1	Model 2	Model 3	Model 1	Model 2	Model 3	Model 1	Model 2	Model 3
	AOR (95% CI)	AOR (95% CI)	AOR (95% CI)	AOR (95% CI)	AOR (95% CI)	AOR (95% CI)	AOR (95% CI)	AOR (95% CI)	AOR (95% CI)
Normal air quality	1.13 (0.89–1.22)		1.06 (1.12–1.47)	0.89 (0.87–1.03)		0.95 (1.07–1.34)	0.95 (0.94–1.12)		0.93 (0.83–0.99)
Poor air quality		0.91 (0.80–1.33)	0.98 (0.93–1.52)		1.08 (1.01–1.29)	1.29 (1.12–1.42)		1.13 (1.06–1.31)	1.24 (1.19–1.46)
Age									
18	Reference	Reference	Reference	Reference	Reference	Reference	Reference	Reference	Reference
19–20	0.69 (0.49–1.17)	0.82 (0.48–1.29)	0.91 (0.51–1.32)	0.77 (0.46–1.49)	1.10 (0.87–1.94)	1.11 (0.78–2.10)	0.87 (0.49–1.84)	0.92 (0.80–1.35)	1.01 (0.48–2.11)
21–24	1.12 (0.50–1.76)	0.88 (0.55–1.59)	1.01 (0.50–1.53)	0.85 (0.62–2.11)	1.43 (0.68–2.71)	1.43 (0.83–2.84)	1.34 (1.10–2.23)	1.03 (0.89–2.06)	1.27 (0.87–3.01)
25–30	1.20 (0.48–2.12)	1.20 (0.52–2.41)	1.17 (0.51–2.28)	0.83 (0.48–2.13)	1.62 (0.89–3.00)	1.39 (0.57–2.90)	1.21 (0.60–2.09)	1.06 (0.75–2.17)	1.73 (1.20–3.01)
Gender									
Female	Reference	Reference	Reference	Reference	Reference	Reference	Reference	Reference	Reference
Male	1.51 (1.03–2.79)	1.49 (1.02–1.94)	1.50 (1.04–2.06)	1.57 (1.20–1.98)	1.74 (1.03–2.21)	1.60 (1.08–2.30)	1.76 (0.96–2.59)	1.69 (1.35–2.27)	1.53 (1.33–1.89)
Monthly living expenses (Yuan)									
1000	Reference	Reference	Reference	Reference	Reference	Reference	Reference	Reference	Reference
1001–2000	1.08 (0.46–1.13)	1.01 (0.48–1.52)	1.06 (0.63–2.05)	0.78 (0.46–2.17)	0.82 (0.48–1.15)	0.87 (0.67–1.12)	1.07 (0.73–1.90)	1.02 (0.79–1.94)	1.07 (0.72–1.89)
2001–3000	1.13 (0.48–1.77)	1.08 (0.79–2.38)	1.12 (0.68–2.93)	0.96 (0.76–2.38)	0.91 (0.46–1.90)	0.76 (0.51–1.30)	0.93 (0.64–0.95)	0.97 (0.87–0.99)	0.98 (0.60–099)
3001	1.22 (1.06–1.98)	1.79 (1.68–2.41)	1.38 (1.09–2.64)	0.84 (0.56–0.95)	1.24 (1.07–1.97)	0.89 (0.68–0.98)	0.92 (0.76–0.98)	1.35 (1.16–2.08)	0.94 (0.90–2.78)
Education									
985 project	Reference	Reference	Reference	Reference	Reference	Reference	Reference	Reference	Reference
211 project	1.34 (0.73–2.98)	1.42 (0.98–2.94)	1.47 (1.09–2.06)	1.28 (0.73–1.70)	1.18 (0.63–2.12)	1.32 (0.86–1.90)	1.24 (0.79–2.08)	1.39 (0.92–2.36)	1.42 (1.03–3.11)
Yi Ben	1.53 (0.68–2.03)	1.56 (0.82–3.16)	1.58 (1.06–1.98)	1.20 (0.81–1.94)	1.03 (0.74–2.36)	1.47 (0.95–1.99)	1.31 (1.23–2.11)	1.25 (1.09–2.64)	1.30 (1.05–3.11)
Er Ben	1.79 (1.11–2.82)	1.69 (1.04–1.94)	1.63 (1.11–2.84)	1.45 (0.90–2.21)	1.42 (0.97–2.07)	1.44 (1.02–2.01)	1.29 (0.57–2.10)	1.41 (0.93–2.30)	1.52 (1.03–3.04)
San Ben	1.77 (1.13–3.01)	1.64 (1.11–3.16)	1.68 (1.09–3.19)	1.42 (0.87–2.30)	1.78 (1.19–2.43)	1.50 (0.97–2.27)	1.45 (1.02–1.95)	1.34 (1.25–3.12)	1.47 (1.27–3.16)
Place of birth									
Rural	Reference	Reference	Reference	Reference	Reference	Reference	Reference	Reference	Reference
Urban	1.45 (1.08–1.98)	1.42 (1.04–2.02)	1.63 (1.03–2.85)	1.60 (1.05–2.98)	1.72 (1.13–2.82)	1.58 (1.28–2.01)	1.67 (1.53–2.42)	1.32 (1.11–2.74)	1.33 (1.02–3.16)
Subjects									
Humanities and Social Sciences	Reference	Reference	Reference	Reference	Reference	Reference	Reference	Reference	Reference
Science	0.96 (0.66–1.39)	0.84 (0.42–1.77)	0.87 (0.69–1.72)	1.01 (0.83–1.94)	1.43 (0.97–2.01)	1.29 (0.85–2.02)	1.31 (0.55–2.07)	1.45 (0.79–2.36)	1.49 (0.67–2.95)
Engineering	1.08 (0.59–1.93)	1.16 (0.57–1.85)	1.16 (0.74–1.95)	0.97 (0.56–1.32)	1.31 (1.02–1.98)	1.47 (1.04–2.21)	1.20 (0.64–2.73)	1.30 (0.87–3.13)	1.45 (0.97–3.12)

**Table 4:** Multivariable associations of psychological well-being operationalised in tertiles.

	Life Satisfaction			Hedonic Unhappiness			CES-D		
	Model 1	Model 2	Model 3	Model 1	Model 2	Model 3	Model 1	Model 2	Model 3
	AOR (95% CI)	AOR (95% CI)	AOR (95% CI)	AOR (95% CI)	AOR (95% CI)	AOR (95% CI)	AOR (95% CI)	AOR (95% CI)	AOR (95% CI)
Normal air quality	1.11 (1.04–1.98)		1.16 (1.08–1.31)	0.89 (0.82–0.95)		0.91 (0.87–0.98)	0.96 (0.81–0.98)		0.93 (0.92–0.99)
Poor air quality		0.97 (0.89–0.99)	0.91 (0.83–0.95)		1.14 (1.03–1.29)	1.11 (0.99–1.23)		1.15 (1.06–1.39)	1.03 (1.02–1.35)
Age									
18	Reference	Reference	Reference	Reference	Reference	Reference	Reference	Reference	Reference
19–20	0.81 (0.46–1.28)	0.88 (0.79–1.26)	1.21 (0.65–2.13)	0.73 (0.64–1.39)	1.02 (0.73–1.67)	1.13 (0.86–1.38)	1.20 (0.73–1.59)	1.39 (1.02–1.67)	1.52 (1.05–1.67)
21–24	0.76 (0.46–1.32)	0.82 (0.51–1.59)	1.17 (0.52–2.17)	0.71 (0.57–1.23)	0.93 (0.64–1.56)	1.04 (0.76–1.47)	1.08 (0.62–1.45)	1.45 (0.64–1.68)	1.37 (0.84–1.86)
25–30	1.12 (0.53–1.46)	1.10 (0.54–1.98)	1.70 (0.96–2.99)	1.02 (0.85–1.34)	1.34 (0.75–2.10)	1.25 (0.82–1.50)	1.25 (0.67–1.62)	1.24 (0.66–1.39)	1.59 (1.02–1.95)
Gender									
Female	Reference	Reference	Reference	Reference	Reference	Reference	Reference	Reference	Reference
Male	1.61 (1.23–1.70)	1.64 (1.32–1.87)	1.70 (1.51–2.02)	1.56 (1.09–2.12)	1.69 (1.34–2.07)	1.73 (1.02–2.73)	2.03 (1.45–2.87)	2.78 (1.32–3.00)	2.93 (2.04–3.38)
Monthly living expenses (Yuan)									
1000	Reference	Reference	Reference	Reference	Reference	Reference	Reference	Reference	Reference
1001–2000	1.26 (0.85–1.91)	1.31 (0.82–1.99)	1.34 (0.64–1.78)	0.92 (0.87–1.95)	0.94 (0.92–2.01)	0.99 (0.81–2.04)	1.75 (0.91–2.43)	2.07 (1.84–2.73)	2.01 (1.74–2.84)
2001–3000	1.43 (0.89–1.67)	1.57 (0.77–2.12)	1.60 (0.84–2.36)	0.97 (0.93–2.06)	0.93 (0.92–2.13)	0.91 (0.94–2.43)	0.93 (0.87–1.89)	0.96 (0.91–0.98)	0.87 (0.83–0.94)
3001	1.58 (0.70–1.84)	1.60 (0.85–2.33)	1.62 (0.91–2.78)	0.83 (0.72–0.99)	0.85 (0.82–0.95)	0.84 (0.81–0.97)	0.81 (0.73–0.97)	0.88 (0.75–0.99)	0.84 (0.78–0.95)
Education									
985 project	Reference	Reference	Reference	Reference	Reference	Reference	Reference	Reference	Reference
211 project	1.58 (0.87–1.80)	1.55 (0.83–1.78)	1.58 (0.99–1.82)	1.43 (0.69–1.65)	1.49 (0.89–1.93)	1.53 (0.61–2.09)	1.44 (0.56–1.75)	1..64 (1.06–2.03)	1.75 (1.05–2.93)
Yi Ben	1.51 (0.94–1.89)	1.49 (0.86–1.63)	1.46 (0.92–1.70)	1.40 (0.96–1.93)	1.53 (0.70–2.07)	1.50 (0.78–2.13)	1.56 (1.39–2.94)	1.57 (1.21–1.95)	1.56 (1.32–2.05)
Er Ben	1.45 (0.89–1.67)	1.42 (0.81–1.32)	1.39 (0.93–1.77)	1.32 (0.67–1.93)	1.43 (0.84–2.01)	1.43 (0.92–2.30)	1.48 (1.04–2.43)	1.69 (1.32–2.07)	1.84 (0.98–2.03)
San Ben	1.42 (0.79–1.84)	1.45 (0.71–1.52)	1.38 (0.75–1.61)	1.29 (0.75–1.68)	1.31 (0.65–2.12)	1.32 (0.67–1.84)	1.67 (1.03–2.05)	1.94 (1.36–3.12)	1.93 (1.02–2.15)
Place of birth									
Rural	Reference	Reference	Reference	Reference	Reference	Reference	Reference	Reference	Reference
Urban	1.36 (0.82–1.56)	1.31 (0.86–1.57)	1.20 (0.74–1.71)	1.73 (1.02–2.57)	1.66 (1.05–2.39)	1.98 (1.05–3.03)	2.18 (1.43–2.94)	1.94 (1.37–2.01)	1.83 (1.43–2.17)
Subjects									
Humanities and Social Sciences	Reference	Reference	Reference	Reference	Reference	Reference	Reference	Reference	Reference
Science	1.48 (0.79–1.83)	1.50 (1.04–1.99)	1.39 (0.75–1.54)	1.59 (0.95–1.87)	1.63 (0.85–1.94)	1.74 (0.94–2.15)	2.08 (1.73–2.87)	1.85 (1.45–2.16)	1.93 (1.46–2.74)
Engineering	1.59 (1.10–2.17)	1.31 (1.15–1.58)	1.42 (1.07–1.90)	1.37 (0.93–1.85)	1.45 (0.79–2.36)	1.56 (0.75–2.13)	1.94 (1.34–2.19)	2.04 (1.38–2.56)	1.68 (1.32–2.10)
